# The nature of food promotions over one year in circulars from leading Belgian supermarket chains

**DOI:** 10.1186/s13690-021-00591-7

**Published:** 2021-05-19

**Authors:** Stefanie Vandevijvere, Iris Van Dam

**Affiliations:** 1 Sciensano (Scientific Institute of Public Health), J.Wytsmanstraat 14, 1050 Brussel, Belgium; 2 INRA (Institut National de la Recherche Agronomique), Paris, France

**Keywords:** Supermarkets, Circulars, Promotions, Ultra-processed foods, Belgium

## Abstract

**Background:**

To examine the proportion of healthier and less healthy food promotions in circulars of major Belgian supermarket chains.

**Methods:**

Food promotions were collected from all circulars over 1 year from the five largest Belgian supermarket chains. Foods promoted were classified according to the World Health Organization Europe nutrient profile model categories and the level and purpose of processing as per the NOVA classification. In addition, promotional characters (i.e. cartoons, licensed characters, celebrities) and premium offers within the promotions were analysed.

**Results:**

In total, 15,271 food promotions were analyzed. The most frequently promoted foods in circulars were processed meat, poultry and fish (11.8%); fresh and frozen fruit, vegetables and legumes (9.5%); soft drinks and sweetened beverages (9.0%); fresh and frozen meat, poultry, fish and eggs (8.6%); cakes, sweet biscuits and pastries (8.1%); ready-made and convenience foods (8.0%); chocolate and sugar confectionery; energy bars and sweet toppings (7.7%) and cheeses (5.7%). About 52.2% of food promotions across all circulars were for ultra-processed foods, with considerable variation across chains (42.9–61.6%).

Promotional characters and premium offers were found within 5.3 and 19.5% of promotions respectively. For all chains, circular covers were healthier compared to entire circulars, with a lower proportion of ultra-processed foods and a higher proportion of fresh fruit and vegetables promoted.

**Conclusions:**

Food promotions in circulars were most frequently for ultra-processed foods, with considerable variation across chains. Circular covers were healthier than entire circulars. Policies to reduce less healthy food promotions could contribute to improving the healthiness of supermarket food purchases.

## Background

Unhealthy diets and overweight and obesity are the key risk factors for the development of diet-related non-communicable diseases (NCDs), such as cardiovascular diseases, type 2 diabetes, and several cancers [9]. Food energy supply per capita has increased in most countries, and these increases were previously found to be sufficient to explain concurrent increases in average population body weight in many countries [24].

In Belgium, dietary risks are the top third contributor to the burden of disease (2016), following tobacco and high blood pressure [13]. Belgians are not meeting most food-based dietary guidelines [4, 23], especially for fruits and vegetables and for limiting energy-dense, nutrient-poor foods. In 2014, only 2.1% of children (3–9 years), 2.4% of adolescents (10–17 years) and 6.6% of adults complied with the recommendations to limit consumption of energy-dense nutrient-poor foods [4]. The usual proportion of daily energy intake from ultra-processed food products was 33.3% for children, 29.2% for adolescents and 29.6% for adults in 2014 [25].

A key driver of unhealthy diets is the obesogenic nature of current food environments, i.e. the relative availability, accessibility and affordability of unhealthy versus healthy foods [21]. Among other food environments, supermarket food environments have the potential to influence diets at a population scale [8, 12]. In Belgium in 2017, 47.8% of packaged food sales were derived from supermarkets, 7.2% from hypermarkets and 15.5% from discounters. For soft drinks these percentages were 56.3, 9.3 and 12.3% respectively [7]. Supermarkets’ marketing techniques have been shown in several countries to significantly influence consumer food purchasing behaviours [3, 5, 10, 11]. Supermarket circulars are one of the common ways of marketing, reaching a big number of consumers [5].

Previous studies in various countries found that such circulars generally contain a high proportion of promotions for less healthy food products [6, 14, 16, 19, 22]. A recent Australian study also found that price promotions were more prevalent and greater in magnitude for less healthy foods than for healthy foods in leading supermarket chains [20].

This study aimed to assess, for the first time, the healthiness of the entire content, as well as the front covers, of supermarket circulars from the leading supermarket chains in Belgium, over 1 year, including the use of marketing techniques, such as promotional characters and premium offers.

## Methods

### Selection of supermarkets

In total 5 supermarkets (including 2 discounters), with a combined market share of 54.2%, based on the 2018 Euromonitor market share data for Belgium, were selected, including the following six companies (and respective market shares): Colruyt (17%), Delhaize (12.4%), Aldi (8.5%), Carrefour Market (6%), Carrefour (4.8%) and Lidl (5.5%). According to Euromonitor 2018 data, these leading supermarket chains together contribute 24.5% to the sales of packaged food products in Belgium through the sales of their own brand products [7].

### Collection of circulars

All weekly or two-weekly (dependent on the supermarket) circulars were collected over a one-year period from May 2019 through to May 2020. All circulars were available in Dutch and were sourced online from each of the supermarket websites. Due to COVID-19 and temporary government measures prohibiting promotions, Colruyt, Delhaize and Carrefour did not publish any folders in the month of April 2020.

### Coding of food promotions

All food promotions in the circulars were manually coded. A sample of promotions was coded by 2 researchers and disagreements were solved, before all the circulars were coded by 1 researcher. A sample of products was checked at the end for verification by the second researcher.

The following variables were captured for all foods promoted: product name, brand name, front cover/inner pages of circular, fresh fruit and vegetables (yes/no), fresh meat and fish (yes/no). All food products on all pages of every circular were coded into one of the 17 categories of the World Health Organization (WHO) Europe nutrient profile model (Table 1). In addition, all food products were coded according to the extent and purpose of food processing using the NOVA classification [17]. The NOVA classification divides foods into four groups:

1. unprocessed or minimally processed foods,
2. processed culinary ingredients,
3. processed foods and
4. ultra-processed foods.

**Table 1:** Food categories included in the WHO Europe nutrient profile model

Group	Name
1	Chocolate and sugar confectionery, energy bars, and sweet toppings and desserts
2	Cakes, sweet biscuits and pastries; other sweet bakery wares, and dry mixes for making such
3	Savoury snacks
4	Beverages
4A	a) Juices
4B	b) Milk drinks
4C	c) Energy drinks (often contain o.a. guarana, taurine, glucuronolactone and vitamins)
4D	d) Other beverages (Soft drinks, sweetend beverages)
5	Edible ices
6	Breakfast cereals
7	Yoghurts, sour milk, cream and other similar foods
8	Cheese
9	Ready-made and convenience foods and composite dishes
10	Butter and other fats and oils
11	Bread, bread products and crisp breads
12	Fresh or dried pasta, rice and grains
13	Fresh and frozen meat, poultry, fish and similar +eggs
14	Processed meat, poultry, fish and similar
15	Fresh and frozen fruit, vegetables and legumes
16	Processed fruit, vegetables and legumes
17	Sauces, dips and dressings

Ultra-processed foods (UPF) are products made mostly or entirely from substances extracted from foods or derived from food constituents with little if any intact food, which often contain flavours, colours and other additives that mimic or intensify the sensory qualities of foods or culinary preparations made from foods [17].

A detailed explanation on the application of the NOVA classification for foods consumed in Belgium has been explained elsewhere [25].

In addition, it was recorded whether the food promotions (excluding those on the food packages) contained any promotional characters or premium offers. Definitions for promotional characters and premium offers were used from the protocol of the International Network for Food and Obesity/NCDs Research Monitoring and Action Support (INFORMAS) [15]. Promotional characters included: cartoon/company owned characters (e.g. M&Ms), licensed characters (e.g. Dora the explorer), famous sport persons/teams, amateur sportspersons, non-sport celebrities (e.g. Jamie Oliver), movie tie-ins (e.g. Shrek), sport events, non-sports/historical events/festivals (including, e.g., Christmas and Saint Nicholas Day), ‘For kids’ e.g. image of a child, ‘great for school lunches’, and awards (e.g. Best Food Award 2014). Premium offers included: game and app downloads, contests, gifts or collectables, buy one and get one free. In addition, since April 2019, the Nutri-Score was adopted by the Minister of Health as a voluntary front-of-pack labeling system in Belgium. We assessed the proportion of food promotions in the circulars that showed the Nutri-Score of the foods promoted.

Promotions for non-food products, alcohol and infant formula were excluded from this study. All advertised products were counted separately, including different sizes of the same product, unless they were exact replicas. Different flavours of a particular product were counted as individual products. A promotion was considered ultra-processed as soon as one of the food items promoted was ultra-processed.

### Data analysis

The percentage of foods promoted within each of the major food categories (out of the total number of foods promoted) according to the WHO Europe nutrient profile model as well as the NOVA classification was calculated for each supermarket chain over a one-year period. We conducted a separate analysis for foods promoted on the front covers of the circulars. We also assessed the percentage of food promotions with premium offers and promotional characters and the percentage of those which displayed the Nutri-Score of the foods promoted. In addition, the results were stratified by season. All analyses were performed using SAS9.4.

## Results

In total 15,271 food promotions were found and analyzed over a one-year period, of which 32.2% for Carrefour (market + hypermarket), 24.9% for Colruyt, 18.4% for Lidl, 12.6% for Delhaize and 11.9% for Aldi. There were about 33.3% of food promotions during autumn, 20.9% during spring, 19.1% during summer and 26.7% during winter. About 4.1% of all food promotions appeared on the front cover of the circulars (data not shown).

The most frequently promoted foods were processed meat, poultry and fish (11.8%); fresh and frozen fruit and vegetables and legumes (9.5%); soft drinks and sweetened beverages (9.0%); fresh and frozen meat, poultry, fish and eggs (8.6%); cakes sweet biscuits and pastries (8.1%); ready-made and convenience foods (8.0%); chocolate and sugar confectionery, energy bars and sweet toppings (7.7%) and cheeses (5.7%). About 52.2% of food promotions across all circulars were for ultra-processed food products. Less than 2% of promotions in the circulars indicated the Nutri-Score of the foods promoted (data not shown).

Promotional characters were not commonly used, only among about 5.3% of promotions, and most of those were for historical events (4.7%). Premium offers were more commonly used, among 19.5% of promotions, and most of these were price reductions when buying multiple items, while about 4.0% were gifts or collectables (data not shown).

### Variations in food promotions across supermarket chains

Considerable variations were found across supermarket chains. For example the proportion of promotions for ultra-processed food products varied from 43 to 62% while the proportion of promotions for fresh fruit and vegetables varied from 4 to 18%.

Delhaize used premium offers most frequently (within 42% of promotions), while Colruyt used those the least frequently (within 1.6% of promotions) (Table 2). When considering the front covers of the circulars, Aldi displayed most frequently promotions for fresh fruit and vegetables (40.8%) while Colruyt displayed such promotions the least frequently (12.5%). Promotions for ultra-processed food products were most frequently displayed by Colruyt (72.2%) while least frequently for Delhaize (10.3%) on the circular front covers. For most supermarket chains, the proportion of food promotions for fresh fruit and vegetables was higher than those for ultra-processed food products on the front covers. However, when considering the entire circulars, this was not the case, as for all supermarket chains, the proportion of promotions for ultra-processed food products was considerably higher than for fresh fruit and vegetables (Table 2).

**Table 2:** Healthiness and power of food promotions in circulars across supermarket chains

	Delhaize	Colruyt	CarrefourHM	CarrefourM	Lidl	Aldi
Entire circular						
%Promotional characters	7.8	0.7	6.4	9.3	6.6	4.0
%Premium offers	41.9	1.6	38.6	31.1	10.1	2.4
% fresh fruit and vegetable promotions	6.8	3.9	7.9	9.9	9.5	17.5
% promotions for ultra-processed foods	52.1	61.6	48.4	45.7	42.9	59.6
Front cover circular						
% promotions on front	5.6	1.9	1.5	10.5	5.5	2.7
% fresh fruit and vegetable promotions	24.3	12.5	21.3	20.5	24.5	40.8
% promotions for ultra-processed foods	10.3	72.2	36.2	19.0	19.4	36.7

*CarrefourHM* Carrefour hypermarket, *CarrefourM* Carrefour market

### Variations in food promotions across weeks and seasons

**Fig. 1:**
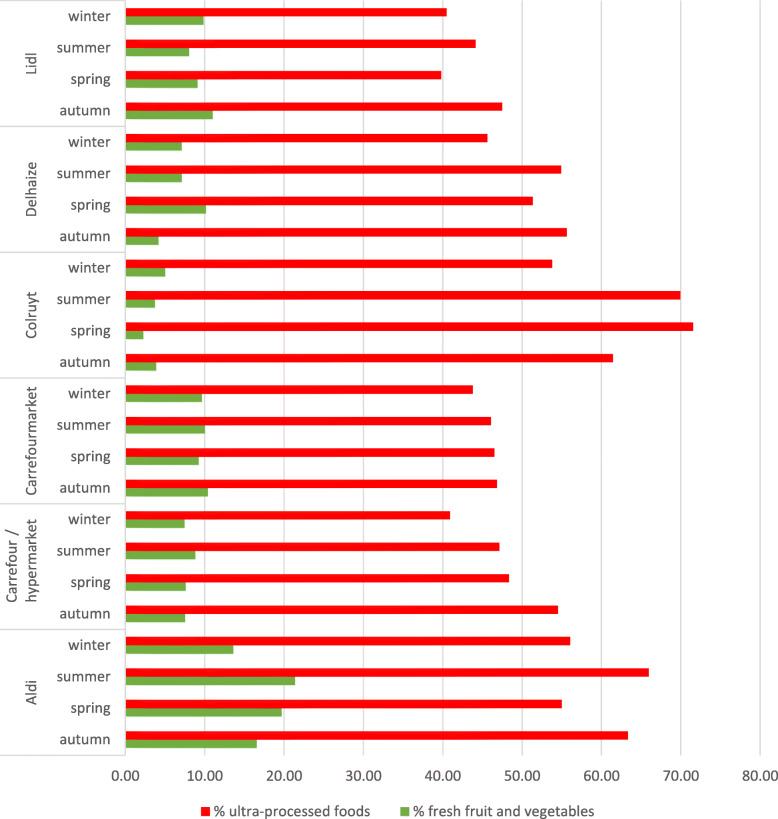
Proportion of promotions for fresh fruit and vegetables and ultra-processed food products by season and supermarket chain

**Fig. 2:**
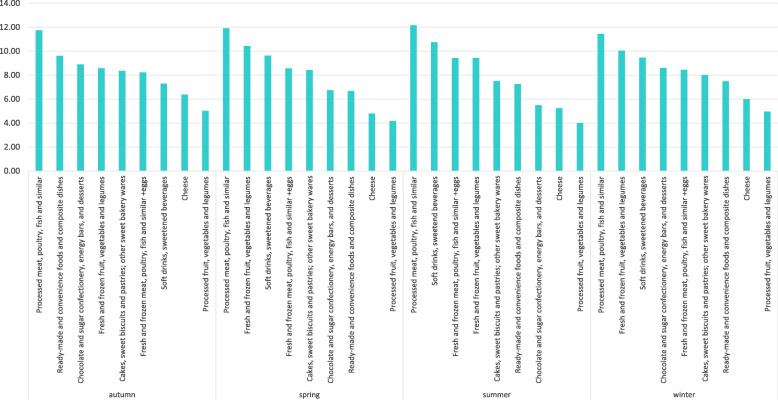
Proportion of promotions for different food categories (including those categories that contribute more than 5% to the total number of promotions) by season

**Fig. 3:**
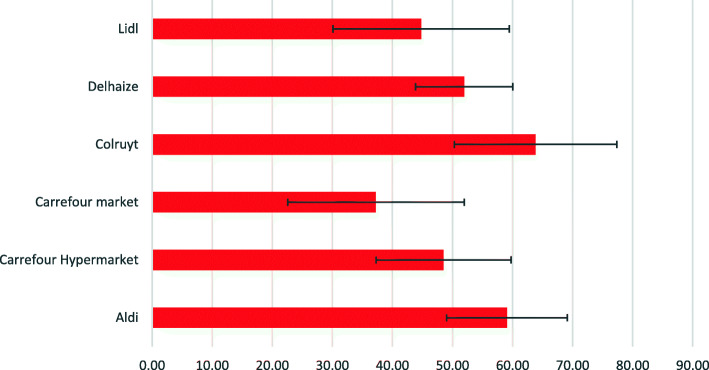
Average (standard deviation) proportion of promotions for ultra-processed foods over a one-year period by supermarket chain

**Fig. 4:**
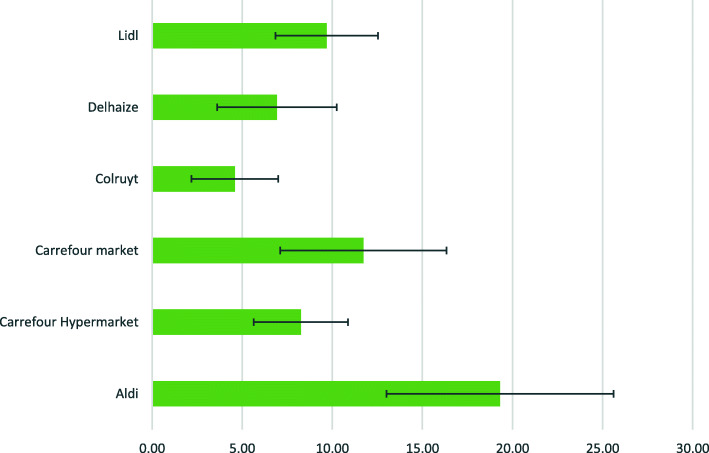
Average (standard deviation) proportion of promotions for fresh fruit and vegetables over a one-year period by supermarket chain

There were large variations for proportion of promotions for ultra-processed food products across weeks over the year: Aldi (59.1 ± 10.1%), Lidl (44.8 ± 14.7%), Colruyt (63.8 ± 13.5%), CarrefourHM (48.5 ± 11.2%), CarrefourM (37.3 ± 14.7%), and Delhaize (52.0 ± 8.1%). Similar results were found for fresh fruit and vegetables (Figure 3 in Appendix 1; Figure 4 in Appendix 2). When considering the different seasons, there were no clear patterns in terms of the proportion of promotions for fresh fruit and vegetables and the proportion of promotions for ultra-processed food products across the different chains (Fig. 1). During all seasons, processed meat and fish were the most frequently promoted food products, followed by soft drinks and sweetened beverages in summer, fresh and frozen fruit and vegetables in winter and spring and ready meals and convenience foods in autumn (Fig. 2).

## Discussion

This study analysed for the first time the food promotions in all circulars from the largest supermarket chains in Belgium over 1 year. In general, more than 50% of promotions were for ultra-processed food products, while less than 10% of promotions were for fresh fruit and vegetables. There were considerable variations in proportion of promotions for ultra-processed food products and fresh fruit and vegetables in circulars across supermarket chains. The proportion of promotions for fresh fruit and vegetables was considerably higher on the front cover of the circulars while the proportion of promotions for ultra-processed food products was considerably lower on the front cover of the circulars compared to the entire circulars for all supermarket chains.

A previous study analysing the contents of supermarket circulars from major supermarket chains in 12 countries internationally over an 8 week period also found a high proportion of promotions for less healthy foods and half of the countries promoting more less healthy than healthy foods inside supermarket circulars. Also in this study, front covers tended to include a much greater proportion of healthy products than the circulars overall [2].

Supermarkets in Belgium have recently made some commitments to improve population nutrition, largely within their role as a manufacturer rather than a retailer. For example, most supermarket chains committed to display the Nutri-Score on the front-of-pack of their own-brand products, even though it is a voluntary measure in Belgium. After 1 year of implementation the Nutri-Score was found on 10% of products in Belgian supermarkets and most of those products were own-brand products from supermarkets [26]. However in the area of food marketing, commitments are weak and generally focus on promoting healthier food options in-store (i.e. like price reductions for Nutri-Score A and B products), rather than restricting promotions for less healthy food options.

Supermarkets generally use different types of promotions, such as price discounts, features and display promotions, or sampling promotions, to market food products to their customers [11] and attract shoppers’ attention [18]. The use of promotions has been shown to increase the sales of the promoted products, which has the potential to affect population dietary patterns through influencing purchasing behaviour [11]. Belgians already consume one third of their daily energy from ultra-processed food products, and those consuming larger proportions of their energy from ultra-processed food products were found to have worse dietary quality [25]. So it is recommended to reduce the marketing for these products.

Strengths of the study include the analysis of circulars over the entire year for the five biggest supermarket chains in Belgium. Limitations include the lack of nutritional composition data to analyse whether promotions are permitted or not according to the WHO Europe nutrient profile model and the lack of data on promotions in-store. Previous research from the UK has shown that, while online nutritional information and prices of products are good proxies of those found in physical stores, this was not necessarily the case for price promotions [1]. In addition, we did not collect information on the size of price promotions in the circulars, while previous research in Australia showed that price promotions on discretionary foods are larger than those on core foods [20]. Such a finding even further increases the urgency for policies to target the healthiness of supermarket environments.

## Conclusion

In conclusion, promotions in supermarket circulars are predominantly for ultra-processed food products in Belgium. Promotions on the covers of the circulars are more frequently for healthy and less frequently for ultra-processed food products compared to the entire circulars.

Stronger commitments from retailers or government policies to reduce the extent of food promotions for less healthy foods could contribute to improving the healthiness of foods purchased from supermarkets in Belgium.

## Data Availability

The datasets used and/or analyzed during the current study are available from the corresponding author on reasonable request.

## Abbreviations

INFORMAS: International Network for Food and Obesity/NCDs Research Monitoring and Action Support
NCDs: Non-communicable diseases
WHO: World Health Organization
